# Icariin protects vertebral endplate chondrocytes against apoptosis and degeneration *via* activating Nrf-2/HO-1 pathway

**DOI:** 10.3389/fphar.2022.937502

**Published:** 2022-09-13

**Authors:** Yuandong Shao, Lei Sun, Guihe Yang, Wenchao Wang, Xiaoyang Liu, Ting Du, Feifei Chen, Xingzhi Jing, Xingang Cui

**Affiliations:** ^1^ Department of Spine Surgery, Shandong Provincial Hospital, Shandong University, Jinan, China; ^2^ Department of Spine Surgery, Binzhou People’s Hospital, Binzhou, China; ^3^ Department of Spine Surgery, Shandong Provincial Hospital Affiliated to Shandong First Medical University, Jinan, China; ^4^ Medical Department, Yidu Cloud (Beijing) Technology Co. Ltd., Beijing, China

**Keywords:** cartilage endplate, intervertebral disc degeneration, icariin, Nrf-2/HO-1, mitophagy, ferroptosis

## Abstract

Cartilage endplate (CEP) plays important roles in the onset and progression of intervertebral disc degeneration (IVDD). Icariin (ICA) is the major active ingredient of *Herba Epimedii* and has various biological activities such as anti-inflammatory and antioxidant, which is used to treat many degenerative diseases. However, the effects and mechanism of ICA on endplate chondrocytes are still unclear. Herein, we studied the effects of ICA on CEP degeneration and elucidated the underlying mechanisms. Endplate chondrocytes were isolated, and TNF-α and TBHP were applied to mimic an IVDD pathological environment. Also, an IVDD mice model was established by transection of bilateral facet joints to investigate the protective effect of ICA *in vivo*. We found that ICA treatment inhibited the chondrocytes apoptosis and the decrease of extracellular matrix production in a dose-dependent manner. Our *in vivo* experiments demonstrated that ICA could ameliorate IVDD development and CEP calcification. We also found that the ICA-activated Nrf-2/HO-1 pathway thus promoted the Parkin-mediated mitophagy process and inhibited chondrocytes ferroptosis, thus alleviated redox imbalance and mitochondrial dysfunction and eventually improved cell survival. Knockdown of Nrf-2 using siRNA reversed the protective effect of ICA on endplate chondrocytes apoptosis and degeneration. In conclusion, our study demonstrated that ICA could protect against CEP degeneration and calcification under IVDD pathological conditions, the associated mechanism may be related to Nrf-2/HO-1-mediated mitophagy activation and ferroptosis inhibition. Our results suggest that ICA may be a potential effective medicine for IVDD prevention and treatment.

## Introduction

Low back pain, which could cause disability and affect people’s daily life, is one of the most common health problems worldwide and results in enormous global burden to public health and social economy. Intervertebral disc degeneration (IVDD) is the leading cause of low back pain (Vergroesen et al., 2015). The intervertebral disc is the cartilaginous tissue located between the vertebral bodies of two adjacent vertebrae and consists of the upper and lower cartilage endplates (CEP), the nucleus pulposus (NP) and the outer annulus fibrosus (AF). The CEP is the main approach for intervertebral disc nutrient supply. Recent studies have demonstrated that cartilage endplate degeneration and calcification can significantly decrease the blood supply status of the intervertebral disc and initiate the IVDD process (Ashinsky et al., 2020). However, the mechanisms of CEP degeneration are not fully elucidated. Also, treatment strategies to early intervene or protect CEP degeneration are still limited. Accordingly, targeting CEP degeneration to improve the intervertebral disc blood supply and inhibit IVDD has become a prospective therapeutic strategy (Li et al., 2010).

The CEP is composed of a layer of hydrated cartilage tissue and chondrocyte is the only cell type in CEP. Various IVDD risk factors such as aging and mechanical stress can induce chondrocytes oxidative stress and low-grade chronic inflammation, which results in elevated reactive oxygen species (ROS) and pro-inflammatory cytokines including IL-1β and TNF-α production (Wang et al., 2016). These mediators could disrupt chondrocytes homeostasis, lead to cell apoptosis, increased cartilage extracellular matrix (ECM) degradation, and CEP calcification. High levels of oxidative stress have been detected in degenerated CEPs, suggesting the involvement of oxidative stress in CEP degeneration (Han et al., 2019). Oxidative stress has been demonstrated playing important roles in osteoarthritis development and cartilage extracellular matrix degradation (Lepetsos and Papavassiliou, 2016; Yang et al., 2020). Recent studies have demonstrated that hydrogen peroxide (H_2_O_2_), a common species of ROS, is responsible for ECM degradation and CEP calcification (Yuan et al., 2019). Overproduction of IL-1β and TNF-α by activated macrophages and chondrocytes could promote chondrocytes apoptosis and ECM degradation, and this also plays important roles in the destruction of endplate homeostasis and chondrocytes apoptosis (Chen et al., 2014). These mediators have overlapping effects, but each is induced by different IVDD risk factors and has its own role in mediating CEP pathogenesis. Therefore, in the present study, we used both H_2_O_2_ and TNF-α to induce CEP degeneration *in vitro* and explored the pathogenesis of IVDD and investigated possible therapeutic approaches.

Many risk factors including aging and mechanical stress could induce oxidative stress and subsequently lead to mitochondrial dysfunction in endplate chondrocytes (Zhang et al., 2009). Mitochondrial dysfunction is characterized by mitochondrial membrane depolarization and mitochondrial ROS overproduction, which could activate the mitochondrial apoptotic pathway and contribute to endplate chondrocyte apoptosis (Song et al., 2021). Mitochondrial autophagy (mitophagy) plays important roles in maintaining mitochondrial homeostasis by clear intracellular damaged mitochondria and decrease mitochondrial ROS production. Recent evidence have found decreased mitophagy marker, Parkin protein in degenerated NP, and that activating mitophagy protects NP and cartilage chondrocytes against mitochondria-dependent apoptosis (Zhang et al., 2018). Apart from mitochondria-dependent apoptosis, ferroptosis is a newly identified iron-dependent programmed cell death and is characterized by mitochondrial contraction, enhanced mitochondrial membrane density, lipid peroxidation, and implication of a unique group of genes. Ferroptosis varies from the other classic cell death processes and recently has been demonstrated to take parts in IVDD by decreasing NP cells viability (Yang et al., 2020). The pathological involvement of ferroptosis in chondrocytes has been well documented in recent studies, and suppression of ferroptosis *via* ferrostatin-1 or inhibiting lipid peroxidation could protect cartilage degeneration (Yao et al., 2021). Moreover, recent studies demonstrated that besides promoting chondrocytes apoptosis and ECM degradation, pro-inflammatory cytokines, IL-1β, and TNF-α could also disrupt cellular iron metabolism homeostasis and increase cellular Fe^2+^ content, thus lead to ferroptosis (Jing et al., 2020). However, the effects of mitophagy and ferroptosis on endplate chondrocyte survival remain largely unknown.

NF-E2-related nuclear factor 2 (Nrf2) is the master sensor of oxidative stress and a regulator of cellular redox homeostasis. The activity of Nrf2 is strictly regulated by Keap1. When the organism is under oxidative stress, Nrf2 is released from the Keap1 binding site and rapidly translocated to the nucleus, then regulate the expression of a variety of genes. The most important downstream target is the regulation of HO-1 expression (Zhang et al., 2020). Previous studies have shown that the Nrf2/HO-1 signaling pathway can regulate the anti-inflammatory and antioxidant properties of chondrocytes and mitigate the progression of osteoarthritis (Khan et al., 2017). It was also found that the Nrf2/HO-1 signaling pathway could mitigate the effects of inflammation and oxidative stress on nucleus pulposus cells in the intervertebral discs, thereby slowing down disc degeneration. In addition, recent studies have reported that the key iron storage proteins ferritin light and heavy chains (FTL/FTH1) and ferroptosis marker, GPX4, are regulated by Nrf2 (17). Targeting Nrf2 to regulate lipid peroxidation and ferroptosis is a potential intervention strategy, but the specific related mechanisms still need to be further explored.

Icariin (ICA) is a flavonoid extract of several plants of the epimedium, a commonly used Chinese herbal medicine with a variety of biological activities: anti-inflammatory, antioxidant, anti-apoptotic, and tumor suppressor. Previous studies have demonstrated the important clinical value of ICA in protecting cartilage chondrocytes in osteoarthritis (Jing et al., 2019). Recent study also indicated that ICA has a protective effect in IVDD through attenuating the oxidative stress-induced mitochondrial dysfunction and cell apoptosis in human NP cells (Hua et al., 2020). Moreover, a variety of signaling pathways, including the Nrf-2/HO-1 and PI3K/AKT pathways, have been proposed to be activated by icariin (Hua et al., 2020). However, the effect and mechanism of ICA on cartilage endplates remain largely unknown. Considering the antioxidant and anti-inflammatory effects of ICA and important roles of cartilage endplates in IVDD, it is necessary to study the protective effect of ICA in cartilage endplate degeneration and its underlying mechanisms.

Therefore, in the present study, we investigated the effects of ICA on cartilage endplate and explored the mechanisms underlying the antioxidant and anti-inflammatory effects of icariin against IVDD pathological condition. Our study may propose new ideas for the treatment of intervertebral disc degeneration.

## Materials and methods

### Reagents

Icariin (purity 98.75%) was purchased from MedChemExpress (MCE, New Jersey, United States) and was dissolved in dimethylsulfoxide (DMSO) for *in vitro* use. Tert-butyl hydroperoxide (TBHP) was obtained from Sigma-Aldrich (St. Louis, MO, United States). Recombinant mouse TNF-α was obtained from R&D systems (Minneapolis, MN, United States).

### Ethics statement

Animal procedures adopted in this study were approved by the Animal Use and Care Committee of Shandong provincial hospital affiliated to Shandong University. Human nucleus pulposus and annulus fibrosus tissues were collected from patients with idiopathic scoliosis undergoing hemivertebrectomy. The collection and treatment of human tissues was approved by the Ethics Committee of Shandong Provincial Hospital affiliated to Shandong University.

### Isolation and culture of murine endplate chondrocytes

Endplate chondrocytes were obtained from 7-day-old C57BL/6J male mice. The cartilage tissue of the vertebral endplate was separated from the spine of mice and minced into pieces. After washed with PBS for three times, 0.25% EDTA trypsin was used to digest the adjacent tissue at 37°C for 30 min. Then washed with PBS and digested with 0.25% Type II collagenase at 37°C for 5 h. Afterward, the primary chondrocytes were collected and cultivated in DMEM/F12 medium with 10% FBS and 100 mg/ml streptomycin sulfate and 100 U/mL penicillin at 37°C. Chondrocytes at passage 1 or 2 were used in the following study.

### Western blotting

After treatment, the cells were washed with PBS twice and lysed with RIPA lysis buffer supplemented with 1% proteinase inhibitor cocktail and 1% phosphatase inhibitor cocktail (CWBIO, Beijing, China) for 60 min on ice. Then lysis solution was collected and centrifuged at 4°C at 12000r/min for 30 min. A bicinchoninic acid test kit was used to determine protein concentration (Solarbio, Beijing, China). Then, on 10% SDS-PAGE gels, protein samples (30 μg) were separated and then electrotransferred to PVDF membranes (Millipore, MA, United States). The membranes were then blocked with 5% non-fat dry milk for 1 h at room temperature and incubated with primary antibodies overnight at 4°C. Antibodies against collagen Type II (#28459-1-AP dilution 1:1000), Nrf2 (#16396-1-AP 1:1000), GAPDH (#10494-1-AP 1:5000), SLC7A11 (#26864-1-AP 1:1000), GPX4 (#67763-1-Ig 1:1000), MMP3 (#17873-1-AP 1:1000), MMP13 (#18165-1-AP 1:1000), PARKIN (#14060-1-AP 1:1000), p65 (#66535-1-Ig 1:1000), IκBα (#10268-1-AP 1:1000), COX2 (#12375-1-AP 1:1000), iNOS (#18985-1-AP 1:1000), Bcl-2 (#26593-1-AP 1:1000), and BAX (#60267-1-Ig 1:5000) were purchased from the Proteintech Group (Chicago, United States). Antibodies against phospho-p65 (#3033 1:1000), cleaved-PARP (#94885 1:1000), pSer139-H2AX (#2577 1:1000), LC3A/B (#4108 1:1000), P62 (#39749 1:1000), and ATG5 (#12994 1:1000) were provided by Cell Signaling Technology Inc. (Beverly, MA, United States). Antibodies against FTH1 (#BM4487 1:1000), HO-1 (#BM4010 1:1000), SOX-9 (#A00177-2 1:1000), COL10A1 (#BA 2023 1:1000), RUNX2 (#PB0171 1:1000), phospho-IκBα (#P001139 1:1000), and cleaved-caspase-3 (#A00334-1 1:1000) were provided by Boster (Wuhan, China). Then the membranes were washed three times in TBST and incubated for 1 hour with peroxidase-conjugated secondary antibody at 25°C (Boster Wuhan, China 1:5000).

Enhanced chemiluminescence reagents (Thermo Fisher) were used to view the membranes, which were then evaluated quantitatively using the BandScan scanner (Bio-Rad, Hercules, CA). The densities of the aforementioned proteins were normalized using GAPDH.

### Reverse transcription quantitative polymerase chain reaction (RT-qPCR)

Total RNA was extracted from CEP chondrocytes using the total RNA extraction kit (Toyobo, Japan), following the manufacturer’s instructions. RNA purity and concentration were measured using a microvolume spectrophotometer (Thermo Fisher Scientific, Logan, UT, United States); 1 μg of total RNA was reverse transcribed into cDNA using First Strand cDNA Synthesis Kit (Toyobo, Osaka, Japan). Real-time PCR was performed under following cycling conditions: 30s of polymerase activation at 95°C, followed by 40 cycles of 95°C for 5s and 60°C for 30s. Sequences of primers for the reference gene (GAPDH) and interested genes are listed as follows: TNFα (F), 5′ -GAC​CCC​TTT​ACT​CTG​ACC​CC- 3′, (R) 5′ -AGG​CTC​CAG​TGA​ATT​CGG​AA-3′; IL-1β (F), 5′ -ACA- GAT​GAA​GTG​CTC​CTT​CCA-3′, (R) 5′ -GTC​GGA​GAT​TCG​TAG​CTG​GA-3′; IL-6 (F), 5′ -TGT​CTT​CCT​CAC​CGA​TTC​CT-3′, (R) 5′ -ACCACCC- GAG​CTC​TGT​CTT​ACT​C-3′; and GAPDH (F) 5′ -AGG​TCG​GTG​TGA​ACG​GAT​TTG-3′, (R) 5′ -TGT​AGA​CCA​TGT​AGT​TGA​GGT​CA-3′;

### siRNA

A particular small interfering RNA (siRNA) targeting the mouse Nrf2 gene was purchased from RiboBio Company (Guangzhou, China) and was then transfected into cells using riboFECTTMCP, according to the manufacturer’s instructions (Guangzhou, China). The siRNA of Nrf2 sequences: sense strand 5′- CAG​GCT​ATC​TCC​TAG​TTC​T-3′.

### Reactive oxygen species (ROS) assay

Levels of CEP chondrocytes ROS production were evaluated using a Reactive Oxygen Species Assay Kit (S0033, Beyotime, Shanghai, China). After treatment, the cells were washed two times with serum‐free DMEM media and incubated with 10 μM DCFH-DA at 37°C for 20 min in darkness. Then the cells were washed three times with serum‐free DMEM media, and fluorescence microscopy (Axio Observer 3; Carl Zeiss) was used to capture the images.

To quantify intracellular ROS level, the cells were collected and washed two times with PBS, and then the cells were treated with 10 μM DCFH-DA at 37°C for 20 min in darkness and washed three times with serum‐free DMEM media. An FACSCalibur flow cytometer was used to assess the mean fluorescence intensity (BD LSRFortessa).

### Annexin V-FITC/PI staining

Annexin V‐FITC/PI apoptosis detection kit (MA0220, Meilunbio, Dalian, China) was used to investigate the anti-apoptotic effect of icariin. After treatment, the cells were washed two times with PBS and stained with annexin V/PI for 20 min at room temperature in darkness. Then, the cells were washed with serum-free DMEM/F12 medium, and an FACSCalibur flow cytometer was used to assess the mean fluorescence intensity. Annexin V^+^/PI^−^ cells were considered as apoptotic cells in the early phase, and annexin V^+^/PI^+^ cells were considered as apoptotic cells in the late phase.

### Determination of mitochondrial membrane potential (MMP), malondialdehyde (MDA), and superoxide dismutase (SOD)

The MMP changes, MDA level and SOD activity after various treatments were measured using corresponding kits. After treatment, the MMP changes of cells were examined with mitochondrial membrane potential kit (C2006, Beyotime, Shanghai, China). Briefly, the cells were washed two times with PBS and treated with JC‐1 working solution, which was made by combining JC‐1 staining fluid (1 ml) with DMEM (1 ml) at 37°C for 20 min in the dark. The cells were then washed two times with JC-1 washing buffer. Fluorescence microscopy was used to view the images (Axio Observer 3; Carl Zeiss). The MDA level was determined using the thiobarbituric acid method and MDA detection kit (S0131S, Beyotime, Shanghai, China). SOD activity was detected using the hydroxylamine method and total SOD detection kit (S0086, Beyotime, Shanghai, China).

### Immunofluorescence

Chondrocytes were seeded in the 24-well plate for COL2, Nrf2, and GPX4 staining. After treatment, the cells were washed twice with PBS and fixed with 4% paraformaldehyde for 30 min at room temperature. Then, the cells were washed with PBS for three times and permeabilized with 0.1% Triton X-100 for 10 min. After blocked with 5% BSA at 37°C for 1 h, the cells were incubated with the primary antibodies against COL2 (1:500), Nrf2(1:200), and GPX4 (1:500) at 4°C overnight. Then, the cells were washed three times with PBS and treated with Cy3-conjugated goat antirabbit secondary antibody (#A0516, Beyotime, Shanghai, China 1:500) in the dark at 37°C for 1 h and 4,6-diamidino-2-phenylindole (DAPI) for 10 min. Fluorescence microscopy was used to capture the images (Axio Observer 3; Carl Zeiss).

To examine the mitophagy process of CEP chondrocytes, the cells were washed two times with PBS and incubated with 200 nM Mito-Tracker Red CMXRos (#C1049B, Beyotime, Shanghai, China 1:500) in the dark at 37°C for 20 min. After washed two times with PBS, the cells were fixed with 4% paraformaldehyde for 20 min at room temperature and permeabilized with 0.1% Triton X-100. The cells were then blocked with 5% BSA at 37°C for 1 h and incubated with primary antibodies against Parkin (1:200), LC3B (#ab192890 Abcam, Cambridge, United Kingdom 1:500) at 4°C overnight. After washed with PBS for three times, the cells were treated with FITC-conjugated goat antirabbit secondary antibody (A0562, Beyotime, Shanghai, China 1:500) in the dark at 37°C for 1 h. Then, the cells were washed three times with PBS and stained with 4,6-diamidino-2-phenylindole (DAPI) for 10 min. Fluorescence microscopy was used to capture the images (Axio Observer 3; Carl Zeiss).

### Animals and the IDD model

All animal procedures were approved by the Animal Care Committee of Shandong Provincial Hospital, Shandong University. Thirty eight weeks old male C57BL/6 mice were used in this study. To establish the IDD model, the L4/5 bilateral facet joints, supra- and interspinous ligaments were transected following a protocol that had previously been published (n = 20). Mice in the sham group received the same procedure exclusive transection of bilateral facet joints, supra-, and interspinous ligaments. Mice in the IDD + ICA group were injected intraperitoneally with ICA (30 mg/kg) dissolved in 80 ul vehicle (10% DMSO, 40% PEG300, 5% Tween-80, and 45% saline) every other day for 12 weeks immediately after the surgery. Mice in the sham and IDD groups were injected intraperitoneally with 80 ul vehicle without ICA (10% DMSO, 40% PEG300, 5% Tween-80, and 45% saline). Mice were euthanized at 12 weeks after surgery, and spinal tissue were collected for micro‐CT, histology, and immunohistochemistry analysis.

### Micro‐computed tomography (CT) analysis

Micro‐computed tomography (CT) (Scanco Viva-CT80, Scanco Medical AG, Basserdorf, Switzerland) was used to evaluate the calcification of upper and lower cartilage endplates of the L4/5 intervertebral disc using a resolution of 13.0 μm, 55 kVp, and 145 μA. The built-in software was used to do data processing and 3D reconstruction. The following parameters were calculated using direct 3D measuring techniques: intervertebral disc height and bone volume/tissue volume (BV/TV).

### Histological staining and immunohistochemistry analysis

After the animals were euthanized, the spinal tissue samples were collected and fixed in 4% paraformaldehyde. After decalcified with 10% EDTA solution for 3 weeks, spinal tissue sections were embedded in paraffin wax and cut into 3 µm thickness in the sagittal plane. To measure cartilage endplates breakdown, 10 successive slides from every 30 µm of spine tissue were stained with safranin O/fast green. The degree of degeneration in the intervertebral discs was rated separately by three persons blind to the experimental protocol using the disc degeneration assessment scoring system developed by Luo et al. (2013). For histochemistry, sections were deparaffinized and rehydrated and then blocked in 5% BSA at 37°C for 30 min. Then sections were incubated with the primary antibody (COL2 1:400, MMP3 1:400, Wuhan, China) (COL-X 1:1000 Abcam, Cambridge, United Kingdom) (Nrf2 1:200, GPX4 1:2000 Proteintech Group Wuhan, China) overnight at 4°C and then followed by secondary antibody (Servicebio, Wuhan, China). The Thermo Fisher Scientific light microscope (IX53; Olympus) was used to capture the images to observe the positive cells.

### Statistical analysis

All experiments were independently performed in triplicate. GraphPad Prism 8.0 software was used to examine all of the data. The data are presented as means with standard deviations. To compare differences between two groups, the Student’s *t*-test was utilized. When examining more than two groups, a one-way analysis of variance (ANOVA) was used to find differences between them, followed by a Tukey test. *p*-values < 0.05 were deemed significant.

## Results

### ICA alleviated cartilage endplate degeneration and IVDD progression *in vivo*

**FIGURE 1:**
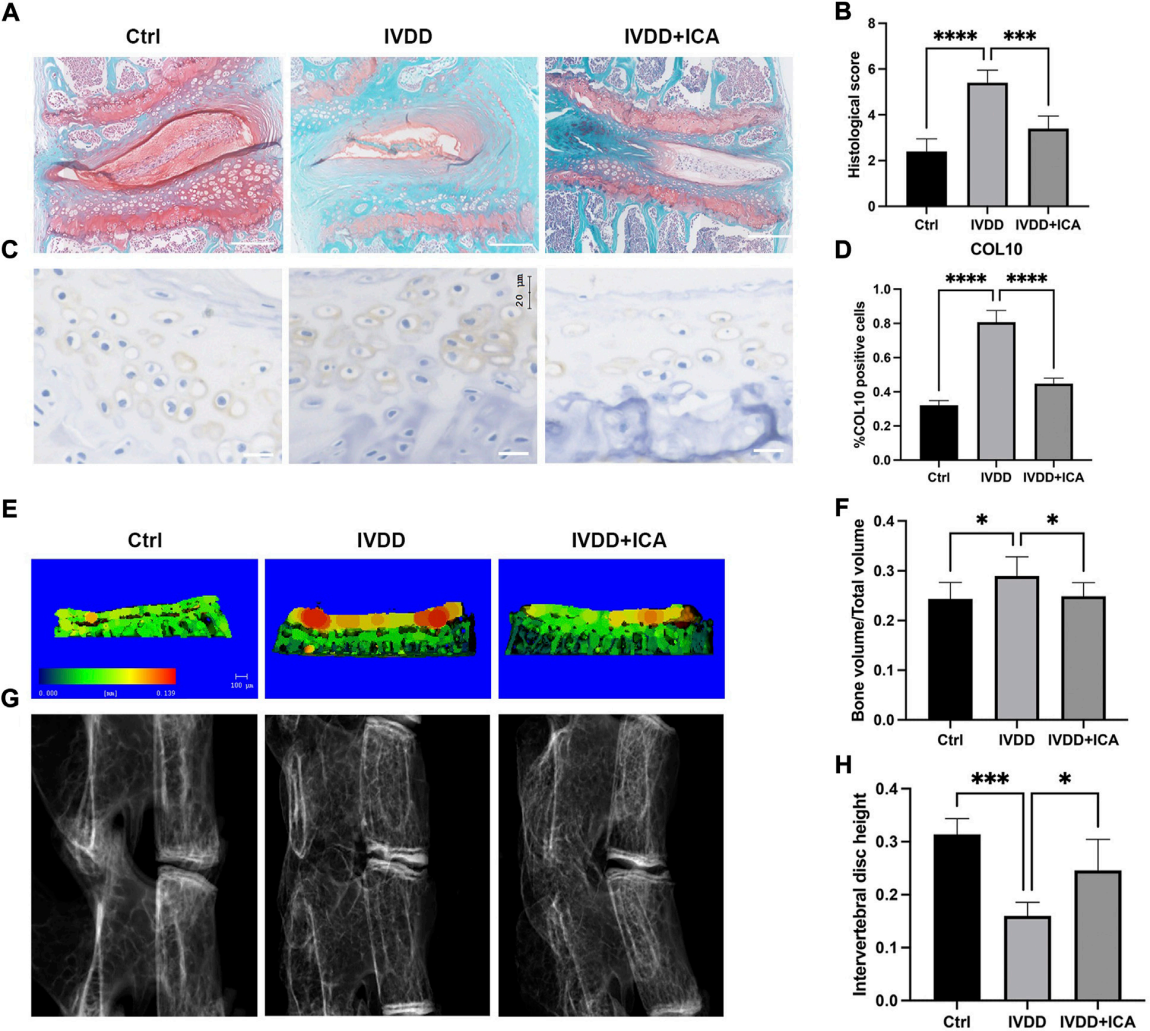
ICA alleviated cartilage endplate degeneration and IVDD progression *in vivo*. **(A)** Safranin O/fast green staining of L4/5 intervertebral discs in the Ctrl, IVDD, and IVDD + ICA groups. Scale bar = 200 μm. **(B)** Histological score of the L4–5 segments of the lumbar spine among the three groups. **(C)** Immunohistochemical staining of COL10 in endplate chondrocytes from each group. Scale bar = 20 μm. **(D)** Ratio of positive cells for COL10 was quantified under a microscope at ×400 magnification using five sections from five mice. **(E)** Micro‐CT analysis of cartilage endplate. Red staining of cartilage endplate means elevated bone mineral density. Scale bar = 100 μm. **(F)** Quantification of cartilage endplate calcification *via* microarchitecture parameters [bone volume per tissue volume (BV/TV)]. **(G–H)** X-ray images of L4/5 segments of lumbar spine among three groups and histomorphometric assessment of intervertebral disc height. The intervertebral disc height was calculated by the average of the anterior, middle, and posterior of intervertebral disc. Data are presented as mean ± SD. *p < 0.05, **p < 0.01, ***p < 0.001, and ****p < 0.0001.

First, an IVDD mice model was established by transection of the L4/5 bilateral facet joints, supra-, and interspinous ligaments to determine whether ICA exerts a protective effect during the progression of IVDD *in vivo*. ICA was intraperitoneally injected immediately after the surgery every other day for 12 consecutive weeks. As shown in Figures 1A, B, safranin O/fast green staining results showed that the IVDD mice model was successfully established, the nucleus pulposus appeared to be reduced in size, and bony tissues that contained bone marrow and mineralized bone were observed in the deep zone of cartilage endplate. Compared to mice in the IVDD group, mice in the ICA treatment group exhibited much more gelatinous NP tissue and few bony tissues in the cartilage endplate. The histological score of the discs in the IVDD + ICA group was significantly lower than that of the IVDD group.

To further investigate the effect of ICA in endplate degeneration and calcification, immunohistochemistry and micro-CT analysis were conducted to examine the chondrocyte hypertrophic and bone mineral density. As shown in Figure 1C, chondrocyte hypertrophic marker, COL10 was significantly upregulated in the IVDD group and this could be reversed by ICA administration. Figures 1E, G showed representative three-dimensional reconstructed micro-CT images of the cartilage endplate and intervertebral disc. As shown in Figures 1F–H, quantification of three-dimensional bone structures revealed that BV/TV in the IVDD + ICA group significantly decreased when compared to the IVDD group. Also the height of intervertebral disc decreased in the IVDD group, and this could be reversed by ICA administration. These results indicated that ICA could alleviate IVDD progression and CEP degeneration.

### ICA suppressed TNF-α induced chondrocytes apoptosis and CEP degeneration

**FIGURE 2:**
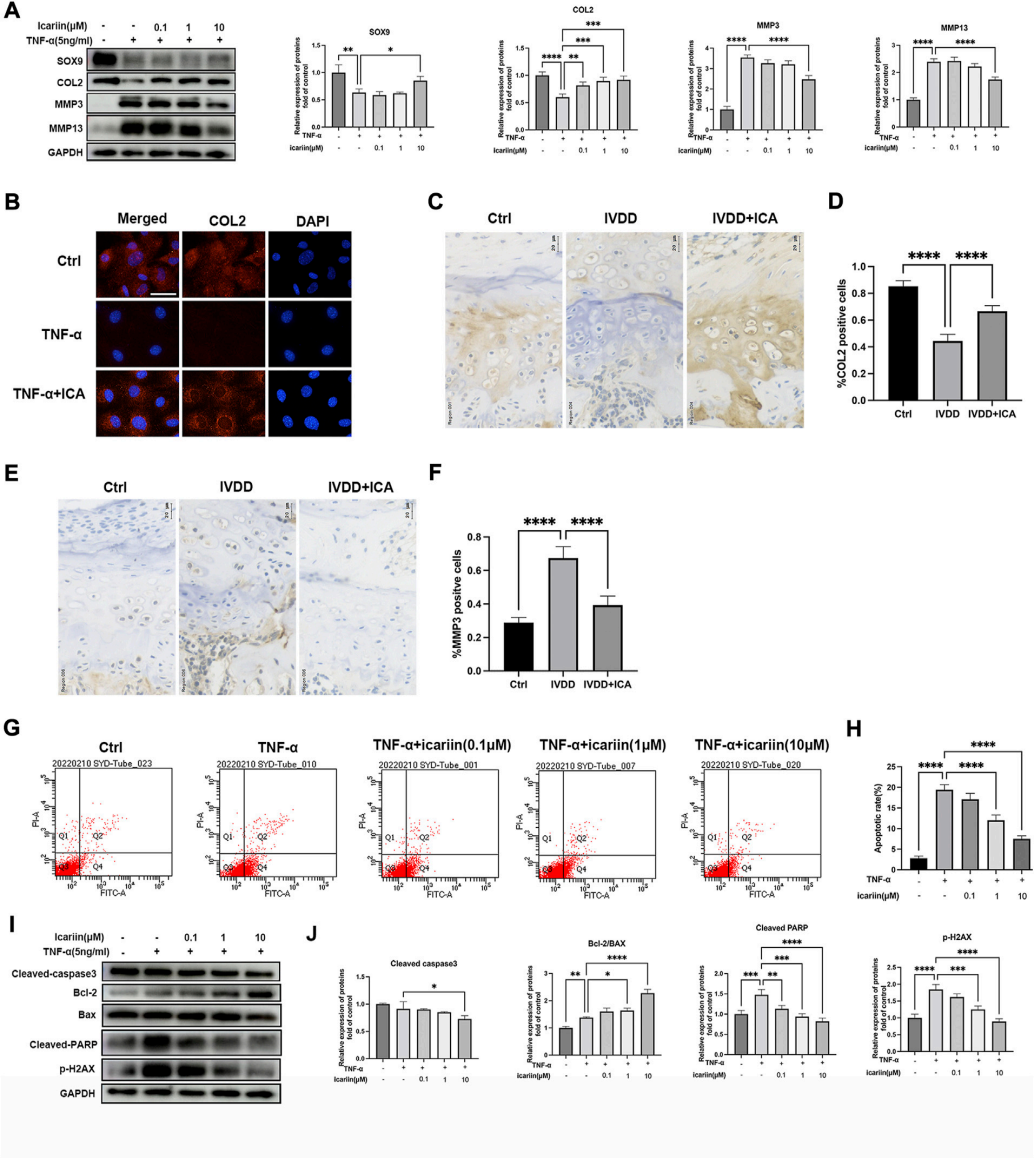
ICA suppressed TNF-α induced chondrocytes apoptosis and CEP degeneration. **(A)** Chondrocytes were treated with TNF-α (5 ng/ml) and ICA (0.1, 1, and 10 μM) for 24 h, and Western blot was conducted to examine the protein levels of COL2, SOX9, MMP3, and MMP13. The band density of COL2, SOX9, MMP3, and MMP13 was quantified and normalized to control. **(B)** Chondrocytes were treated with TNF-α (5 ng/ml) with or without ICA (10 μM) for 24 h, and immunofluorescence staining was conducted to examine the expression of COL2 (red). Scale bar = 20 µm. **(C,E)** Immunohistochemistry for COL2 and MMP3 in the Ctrl, IVDD, and IVDD + ICA groups. Scale bar = 20 μM. **(D,F)** The ratio of positive cells for COL2 and MMP3 in endplate of the Ctrl, IVDD, and IVDD + ICA groups. **(G,H)** Chondrocytes were treated with TNF-α (5 ng/ml) and ICA (0.1, 1, 10 μM) for 24 h, and annexin V-FITC/PI flow cytometric analysis was conducted to measure endplate chondrocyte apoptosis. Annexin V^+^/PI^−^ cells were considered as apoptotic cells in the early phase and annexin V^+^/PI^+^ cells were considered as apoptotic cells in the late phase. **(I–J)** Chondrocytes were treated with TNF-α (5 ng/ml) and ICA (0.1, 1, and 10 μM) for 24 h, and Western blot was conducted to examine the protein levels of cleaved-caspase-3(CC3), Bax, Bcl-2, cleaved-PARP, and pSer139-H2AX. The band density was quantified and normalized to control. All experiments were repeated three times independently. Data are presented as mean ± SD. ∗*p* < 0.05, ∗∗*p* < 0.01, ∗∗∗*p* < 0.001, and ∗∗∗∗*p* < 0.0001.

The pathological progression of IVDD is complex and multifactorial. Various risk factors induced pro-inflammatory cytokines excretion, and oxidative stress played the most important roles in IVDD progression. In the present study, TNF-α was used to mimic the endplate osteochondritis inflammation environment *in vitro*. As shown in Figure 2A, Western blot analysis revealed that 5 ng/ml TNF-α significantly promoted matrix metalloproteinase MMP3 and MMP13 expression, inhibiting chondrogenic differentiation markers, SOX9 and COL2 expressions, and this could be reversed by ICA co-treatment. Our results also showed that the protective effect of ICA was dose-dependent; 10 μM ICA exhibited the most significant protective effect in inhibiting TNF-α induced ECM degradation. Similar results were obtained by immunofluorescence assay that ICA co-treatment reversed the decrease of COL2 induced by TNF-α (Figure 2B). Our *in vivo* experiments also demonstrated that ICA administration significantly promoted COL2 expression (Figures 2C,D) and inhibited MMP3 expression (Figures 2E,F) in endplate cartilage. Chondrocyte is the only cell type in cartilage endplate; flow cytometric analysis was then conducted to assess chondrocyte apoptotic rates using annexin V-FITC/PI staining. TNF-α increased chondrocyte apoptosis compared with the control group, while ICA co-treatment could inhibit TNF-α induced apoptosis (Figures 2G,H). Furthermore, similar results were obtained by Western blot assay that ICA inhibited TNF-α induced cleaved-caspase 3 expression and DNA damage markers, cleaved-PARP and γH2AX, promoted the ratio of Bcl-2/Bax, and thus inhibited mitochondrial apoptotic pathway activation. Taken together, these results indicated that ICA protected against TNF-α induced ECM degradation and chondrocytes apoptosis.

### ICA suppressed oxidative stress induced ECM degradation and chondrocytes apoptosis

**FIGURE 3:**
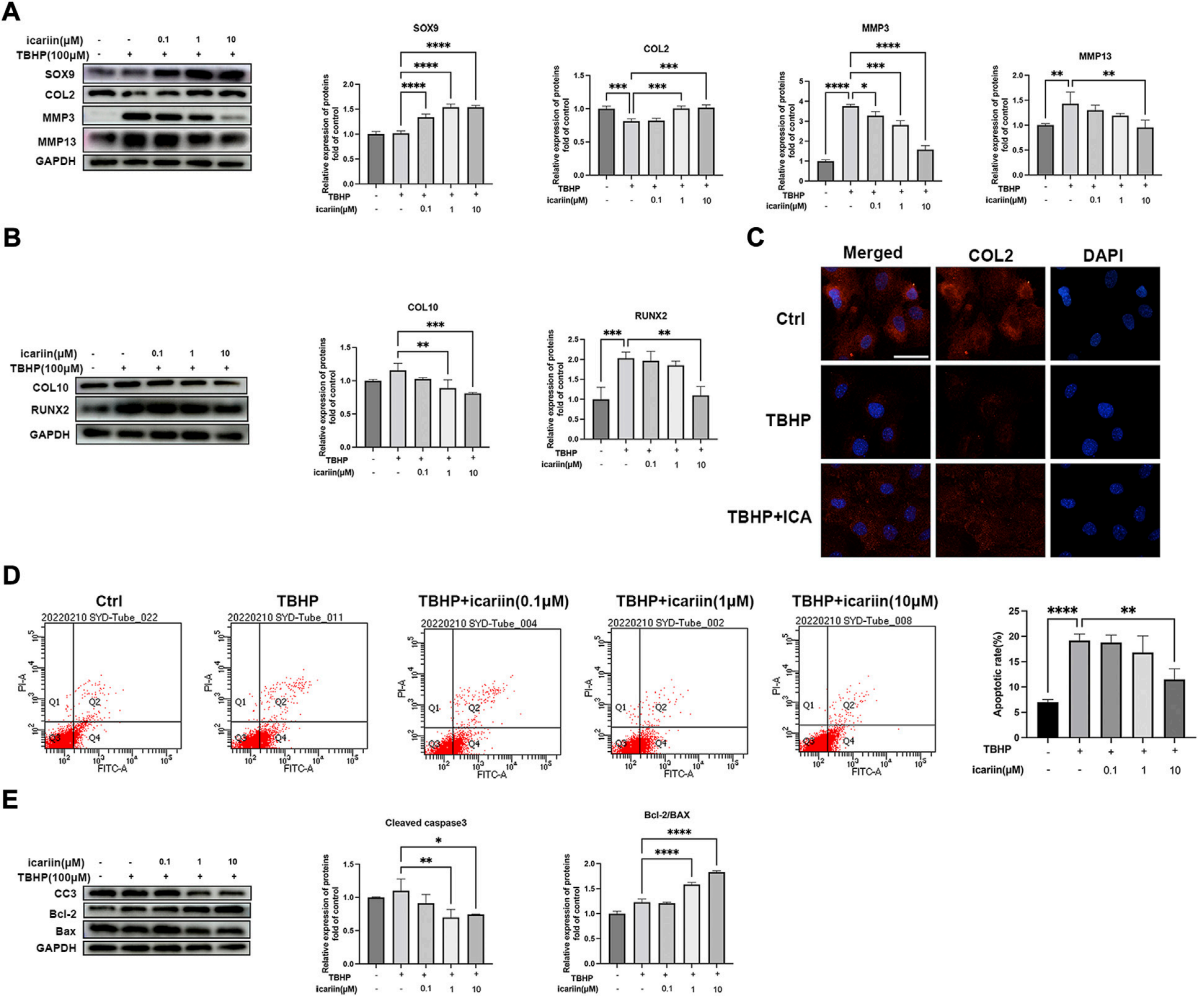
ICA suppressed oxidative stress induced ECM degradation and chondrocytes apoptosis. **(A)** Chondrocytes were treated with TBHP (100 μM) and ICA (0.1, 1, 10 μM) for 24 h, and Western blot was conducted to examine the protein levels of COL2, SOX9, MMP3, and MMP13. The band densities of COL2, SOX9, MMP3, and MMP13 were quantified and normalized to control. **(B)** Chondrocytes were treated with TBHP (100 μM) and ICA (0.1, 1, and 10 μM) for 24 h, and Western blot was conducted to examine the protein levels of COL10 and RUNX2. The band densities of COL10 and RUNX2 were quantified and normalized to control. **(C)** Chondrocytes were treated with TBHP (100 μM) with or without ICA (10 μM) for 24 h, and immunofluorescence staining was conducted to examine the expression of COL2 (red). Scale bar = 20 µm. **(D)** Chondrocytes were treated with TBHP (100 μM) and ICA (0.1, 1, and 10 μM) for 24 h, and annexin V-FITC/PI flow cytometric analysis was conduct to measure endplate chondrocyte apoptosis. **(E)** Chondrocytes were treated with TBHP (100 μM) and ICA (0.1, 1, and 10 μM) for 24 h, and Western blot was conducted to examine the protein levels of cleaved-caspase-3 (CC3), Bax, and Bcl-2. The band density of cleaved-caspase-3(CC3), Bcl-2/BAX ratio was quantified and normalized to control. All experiments were repeated three times independently. Data are presented as mean ± SD. ∗*p* < 0.05, ∗∗*p* < 0.01, ∗∗∗*p* < 0.001, and ∗∗∗∗*p* < 0.0001.

H_2_O_2_ can lead to chondrocyte oxidative stress, with mitochondrial dysfunction and altered MMP levels, which further promote matrix degrading enzymes expression and activate the mitochondrial apoptotic pathway (Song et al., 2021). As shown in Figures 3A, B, D, E, ICA also reversed TBHP-induced CEP chondrocytes degeneration and apoptosis. Similar results were obtained by immunofluorescence assay that ICA co-treatment reversed the decrease of COL2 induced by TBHP (Figure 3C). These data indicated that ICA suppressed the oxidative stress induced ECM degradation, hypertrophic change, and apoptosis in chondrocytes.

### ICA suppressed TNF-α and TBHP induced inflammatory response, oxidative stress, and mitochondrial dysfunction

**FIGURE 4:**
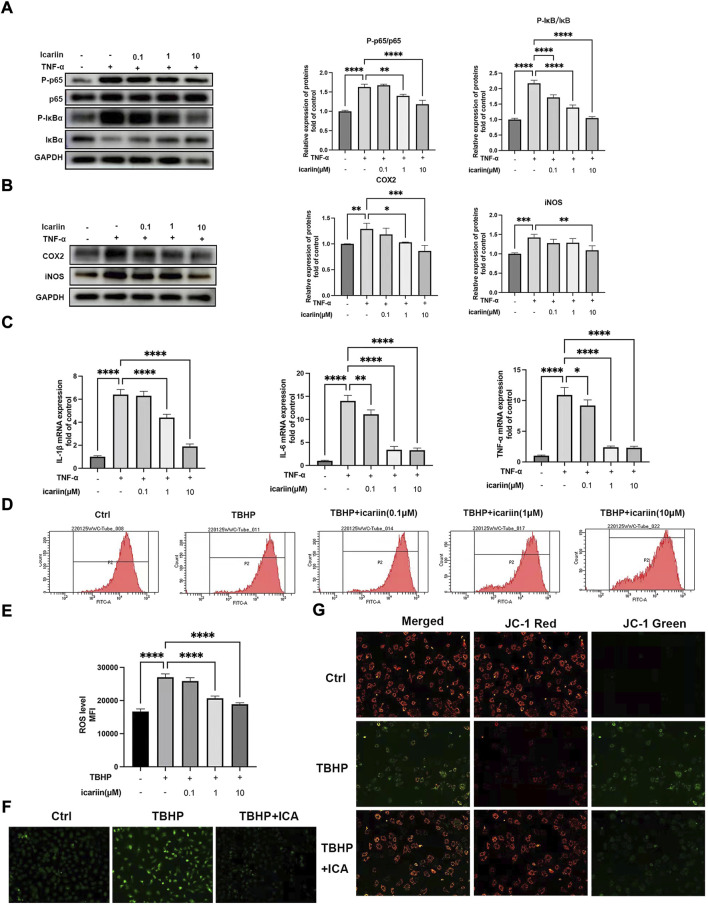
ICA suppressed TNF-α and TBHP-induced inflammatory response, oxidative stress, and mitochondrial dysfunction. **(A)** Chondrocytes were treated with TNF-α (5 ng/ml) and ICA (0.1, 1, and 10 μM) for 24 h, and Western blot was conducted to examine the protein levels of P-p65, p65, P-IκB, and IκB. The ratio of P-p65/p65 and P-IκB/IκB was quantified and normalized to control. **(B)** Chondrocytes were treated with TNF-α (5 ng/ml) and ICA (0.1, 1, and 10 μM) for 24 h, and Western blot was conducted to examine the protein levels of COX2 and iNOS. The band densities of COX2 and iNOS were quantified and normalized to control. **(C)** RT-PCR was used to detect the expression of cytokines including IL-1β, IL-6, and TNFα. **(D–E)** Intracellular ROS levels in the endplate chondrocytes were detected using the DCFH-DA and measured by flow cytometry. **(F)** Representative fluorescence microscopy photomicrographs of intracellular ROS in endplate chondrocytes. Green fluorescence indicates the intracellular ROS production. **(G)** Representative fluorescence microscopy photomicrographs of mitochondrial membrane potential (MMP) after incubating with JC‐1. Red fluorescence was emitted by JC‐1 aggregates in healthy mitochondria with polarized inner mitochondrial membranes, whereas green fluorescence was emitted by cytosolic JC‐1 monomers, indicating MMP collapse. Merged images indicated colocalization of JC‐1 aggregates and monomers. All experiments were repeated three times independently. Data are presented as mean ± SD. ∗*p* < 0.05, ∗∗*p* < 0.01, ∗∗∗*p* < 0.001, and ∗∗∗∗*p* < 0.0001.

NF-κB plays important roles in TNF-α-mediated inflammation and cell apoptosis (Hayden and Ghosh, 2014). We next investigated whether ICA could suppress the NF-κB pathway. As shown in Figure 4A, ICA treatment suppressed TNF-α-induced NF-κB activation, with decreased P-p65/p65 and P-IκB/IκB ratio. Our results also showed that ICA reduced TNF-α induced upregulation of inflammation mediators, iNOS and COX2, and inflammatory cytokines, TNF-αIL-6 and IL-1β (Figures 4B,C). These results indicated that ICA could modulate the TNF-α-induced inflammatory response. Oxidative stress can lead to mitochondrial dysfunction and altered MMP levels, which further stimulates ROS overproduction, forming a vicious cycle. We next investigate whether ICA could protect against TBHP-induced ROS production and mitochondrial dysfunction. Flow cytometry analysis showed that 100 μM TBHP induced marked ROS production and this could be inhibited by ICA treatment (Figures 4D,E). DCFH-DA staining showed that ICA treatment significantly suppressed green fluorescence intensity, representing a decrease in TBHP-induced elevated ROS production (Figure 4F). Alteration of MMP indicates mitochondrial dysfunction. An increase in the fluorescence intensity of green JC‐1 monomers compared with red JC‐1 aggregates was observed in the TBHP group, suggesting a significant reduction of MMP induced by TBHP. However, ICA treatment repressed the changes of MMP with reduced green JC‐1 monomers and increased red JC‐1 aggregates. These results indicated that ICA suppressed TNF-α and TBHP induced endplate chondrocytes inflammatory response, oxidative stress, and mitochondrial dysfunction.

To further investigate the protective effect of ICA in IVDD development and the specific role of ICA in intervertebral disc tissues, human nucleus pulposus cells and annulus fibrosus cells were isolated, and the effect of ICA in TBHP-induced ECM degradation, and apoptosis was examined. We found that ICA also protected nucleus pulposus cells against TBHP-induced ECM degradation and apoptosis (Supplementary Figure S1), these results further demonstrated the effect of ICA in alleviating IVDD progression. No significant protective effect of ICA in annulus fibrosus was observed, although ICA slightly inhibited TBHP-induced apoptosis (Supplementary Figure S2). Also CEP chondrocytes from IVDD model mice were isolated and treated with ICA, endplate chondrocytes from normal mice were isolated as control. As shown in Supplementary Figure S3, we found that endplate chondrocytes from IVDD model mice exhibited lower expressions of SOX9 and COL2 and higher expression of MMP13, which was consistent with our immunohistochemistry results. ICA treatment decreased the expression of MMP3, MMP13, and cleaved-caspase-3, promoted the expression of SOX9, COL2, and the ratio of Bcl-2/BAX of chondrocytes from IVDD model mice. These results further demonstrated the protective effect of ICA in inhibiting endplate degeneration and IVDD development.

### ICA activated chondrocytes Nrf-2/HO-1 pathway and promoted mitophagy to maintain chondrocytes homeostasis under pathological conditions

**FIGURE 5:**
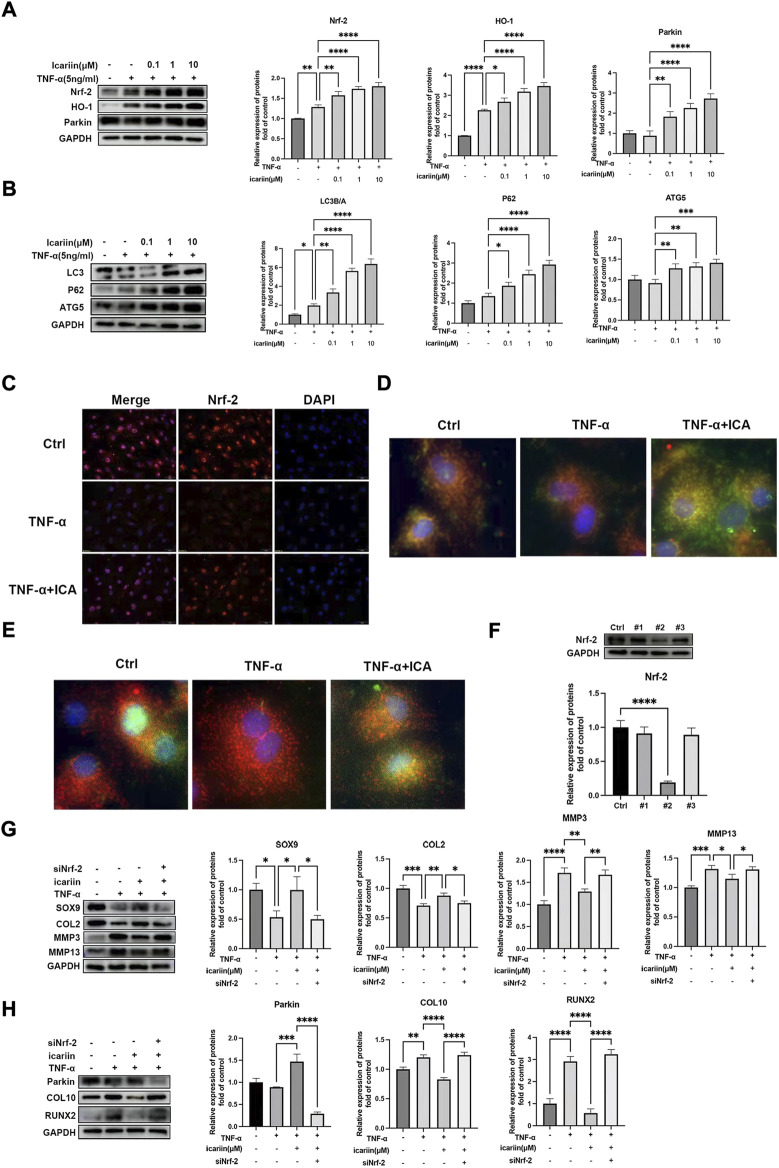
ICA protected against TNF-α induced endplate chondrocytes ECM degradation and apoptosis *via* activating the Nrf-2/HO-1 pathway. **(A–B)** Chondrocytes were treated with TNF-α (5 ng/ml) and ICA (0.1, 1, and 10 μM) for 24 h, and Western blot was conducted to examine the protein levels of Nrf-2, HO-1, Parkin, LC3A/B, P62, and ATG5. The band densities of Nrf-2, HO-1, Parkin, the ratio of LC3B/A, P62, and ATG5 were quantified and normalized to control. **(C)** Chondrocytes were treated with TNF-α (5 ng/ml) with or without ICA (10 μM) for 24 h and immunofluorescence staining of Nrf-2 was conducted. Scale bar = 20 µm. **(D)** Chondrocytes were treated with TNF-α (5 ng/ml) with or without ICA (10 μM) for 24 h and immunofluorescence staining was conducted to examine the expression and localization of Parkin (green) and mitochondria (red). **(E)** Chondrocytes were treated with TNF-α (5 ng/ml) with or without ICA (10 μM) for 24 h and immunofluorescence staining was conducted to examine the expression and localization of LC3B (green) and mitochondria (red). **(F)** Nrf-2 siRNA was synthesized, and transfection efficiency was evaluated by detecting Nrf2 expression using Western blotting. **(G–H)** Chondrocytes were transfected with Nrf-2 siRNA, and then treated with TNF-α and ICA for 24 h. Protein levels of SOX9, COL2, MMP3, MMP13, COL10, RUNX2, and Parkin were determined by Western blot. The band density was quantified and normalized to control. All experiments were repeated three times independently. Data are presented as mean ± SD. ∗*p* < 0.05, ∗∗*p* < 0.01, ∗∗∗*p* < 0.001, and ∗∗∗∗*p* < 0.0001.

The Nrf-2/HO-1 pathway plays pivotal roles in cellular redox. Recent studies indicated that Nrf-2 could directly modulate cellular mitophagy *via* regulating Parkin protein expression (Xiao et al., 2017). Therefore, we next investigated whether ICA could activate the Nrf-2/HO-1 pathway and promote the endplate chondrocyte mitophagy process. As shown in Figures 5A,B, TNF-α slightly promoted Nrf-2 and its downstream cytoprotective genes including HO-1 and Parkin. ICA treatment led to notably increased protein levels of Nrf-2 and its downstream HO-1, Parkin, LC3B, P62, and ATG5 proteins expression. Immunofluorescence staining of Nrf-2 obtained similar results that ICA significantly promoted Nrf-2 protein expression and nucleus translocation (Figure 5C). Mitophagy play important roles in maintaining cellular mitochondrial function and redox homeostasis. The co-localization of autophagosome and Parkin with mitochondria after continuous ICA stimulation suggested that chondrocyte mitophagy was activated (Figure 5D). As shown in Figure 5E, the formation of autophagosomes was increased following ICA treatment with increased immunostaining of microtubule-associated proteins 1B light chain 3B (LC3B). To investigate whether ICA exerted its protective effect through the Nrf-2/HO-1 pathway, Nrf-2 siRNA was synthesized and transfected into endplate chondrocytes (Figure 5F). As shown in Figures 5G–H, knockdown of Nrf-2 inhibited ICA-induced Parkin upregulation, indicating that the ICA-induced mitophagy process was partly Nrf-2-dependent. In addition, knockdown of Nrf-2 abrogated ICA mediated inhibition of TNF-α induced MMP3, MMP13, COL10, RUNX2, and upregulation of SOX9 and COL2.

**FIGURE 6:**
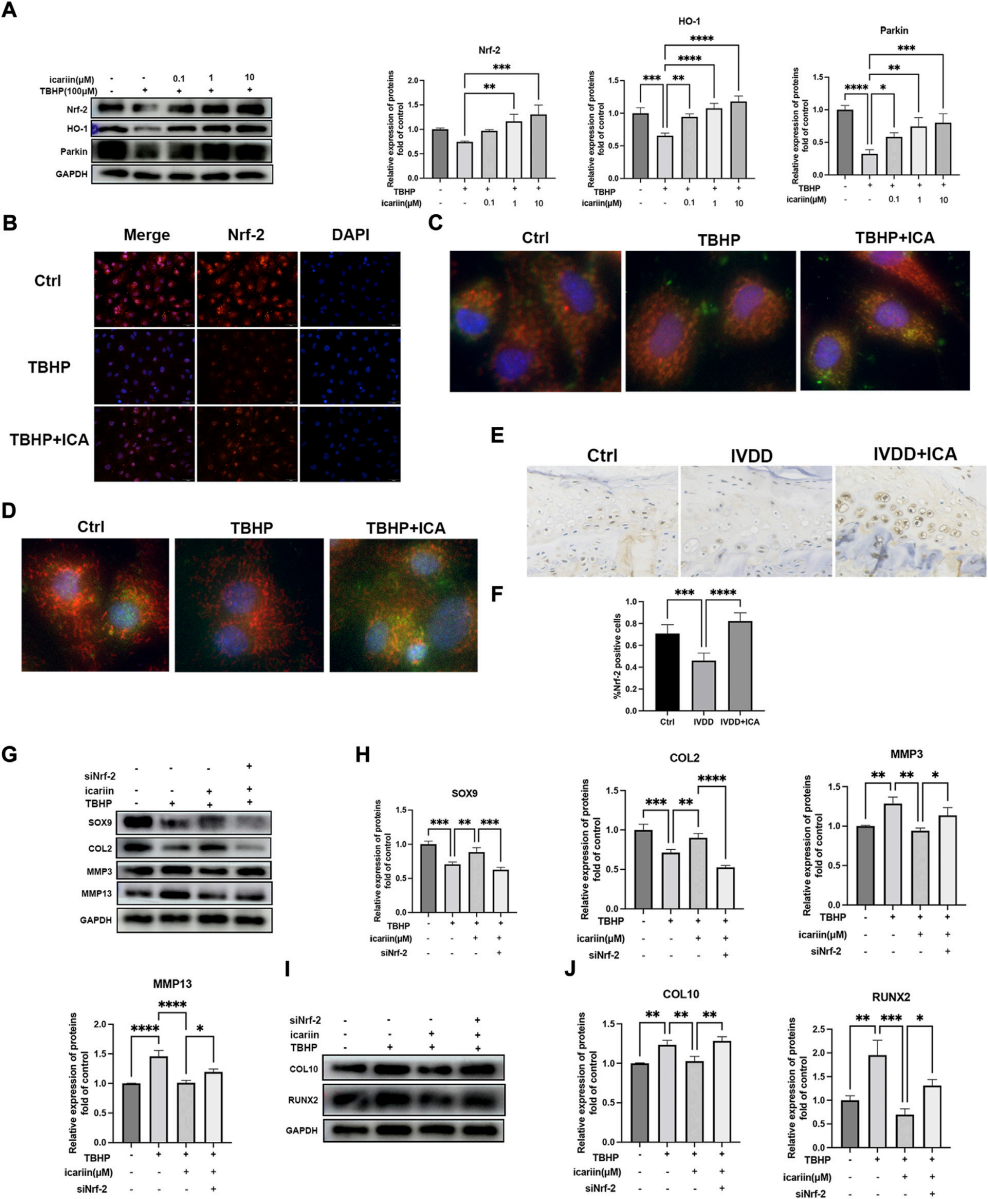
ICA protected against TBHP induced endplate degeneration and chondrocytes apoptosis *via* activating the Nrf-2/HO-1 pathway. **(A)** Chondrocytes were treated with TBHP (100 μM) and ICA (0.1, 1, and 10 μM) for 24 h, and Western blot was conducted to examine the protein levels of Nrf-2, HO-1, and Parkin. The band densities of Nrf-2, HO-1, and Parkin were quantified and normalized to control. **(B)** Chondrocytes were treated with TBHP (100 μM) with or without ICA (10 μM) for 24 h and immunofluorescence staining of Nrf-2 was conducted. Scale bar = 20 µm. **(C)** Chondrocytes were treated with TBHP (100 μM) with or without ICA (10 μM) for 24 h and immunofluorescence staining was conducted to examine the expression and localization of Parkin (green) and mitochondria (red). **(D)** Chondrocytes were treated with TBHP with or without ICA (10 μM) for 24 h, and immunofluorescence staining was conducted to examine the expression and localization of LC3B (green) and mitochondria (red). **(E)** Immunohistochemical staining of Nrf-2 in endplate chondrocytes from the Ctrl, IVDD, and IVDD + ICA groups. Scale bar = 20 μm. **(F)** Ratio of positive cells for Nrf-2 were quantified under a microscope at ×400 magnification using five sections from five mice. **(G–J)** Chondrocytes were transfected with Nrf-2 siRNA, and then treated with TBHP and ICA for 24 h. Protein levels of SOX9, COL2, MMP3, MMP13, COL10, and RUNX2 were determined by Western blot. The band density was quantified and normalized to control. All experiments were repeated three times independently. Data are presented as mean ± SD. ∗*p* < 0.05, ∗∗*p* < 0.01, ∗∗∗*p* < 0.001, and ∗∗∗∗*p* < 0.0001.

We next investigated the role of Nrf-2/HO-1 in TBHP-induced oxidative stress. As shown in Figure 6, similar results were obtained that ICA significantly activated the Nrf-2/HO-1-mediated mitophagy process, *in vivo* expression of Nrf-2 in endplate was also investigated, and immunohistochemistry results showed that mice in the IVDD + ICA group exhibited significant increased Nrf-2 expression in endplate compared to mice in the IVDD group (Figures 6E,F). Knockdown of Nrf-2 abrogated ICA mediated inhibition of TBHP induced MMP3, MMP13, COL10, RUNX2, and upregulation of SOX9 and COL2. These results indicated that the Nrf-2/HO-1 mediated anti-oxidant effect and mitophagy process play important roles in the protective effect of ICA in cartilage endplate degeneration.

### ICA inhibited endplate chondrocyte ferroptosis under pathological conditions *via* activating Nrf-2

**FIGURE 7:**
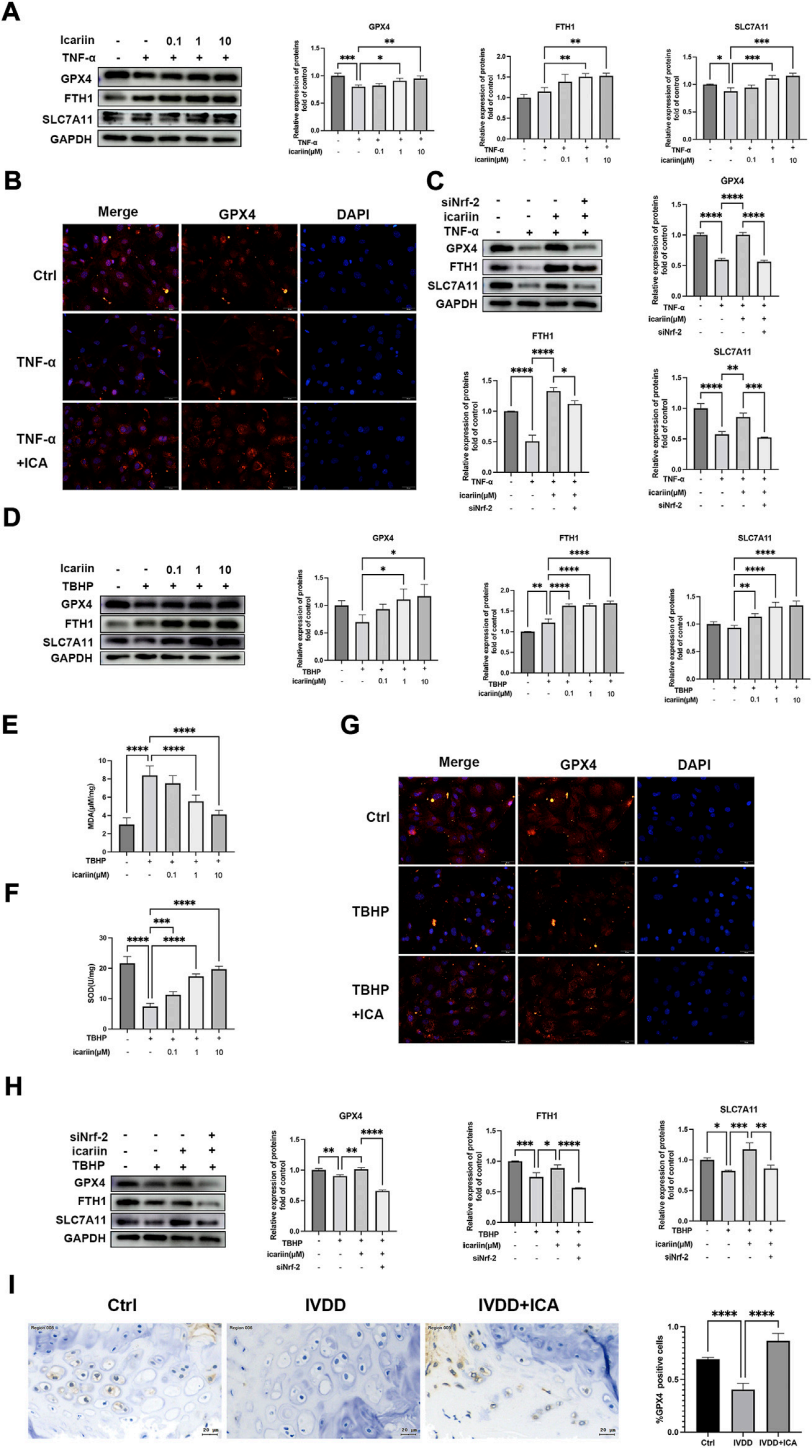
ICA inhibited endplate chondrocyte ferroptosis under pathological conditions *via* activating Nrf-2. **(A)** Chondrocytes were treated with TNF-α (5 ng/ml) and ICA (0.1, 1, 10 μM) for 24 h, and Western blot was conducted to examine the protein levels of GPX4, FTH1, and SLC7A11. The band densities of GPX4, FTH1, and SLC7A11 were quantified and normalized to control. **(B)** Chondrocytes were treated with TNF-α (5 ng/ml) with or without ICA (10 μM) for 24 h, and immunofluorescence staining was conducted to examine the expression and localization of GPX4. Scale bar = 20 µm. **(C)** Chondrocytes were transfected with Nrf-2 siRNA, then treated with TNF-α and ICA for 24 h. Protein levels of GPX4, FTH1, and SLC7A11 were determined by Western blot. The band density was quantified and normalized to control. **(D)** Chondrocytes were treated with TBHP (100 μM) and ICA (0.1, 1, and 10 μM) for 24 h, and Western blot was conducted to examine the protein levels of GPX4, FTH1, and SLC7A11. The band densities of GPX4, FTH1, and SLC7A11 were quantified and normalized to control. **(E–F)** The level of MDA and the activities of SOD were detected. **(G)** Chondrocytes were treated with TBHP (100 μM) with or without ICA (10 μM) for 24 h, and immunofluorescence staining was conducted to examine the expression and localization of GPX4. Scale bar = 20 µm. **(H)** Chondrocytes were transfected with Nrf-2 siRNA, then treated with TBHP and ICA for 24 h. Protein levels of GPX4, FTH1, and SLC7A11 were determined by Western blot. The band density was quantified and normalized to control. **(I)** Immunohistochemical staining of GPX4 in endplate chondrocytes from the Ctrl, IVDD and IVDD + ICA groups. Scale bar = 20 μm. The ratio of positive cells for GPX4 in endplate of the Ctrl, IVDD, and IVDD + ICA groups. All experiments were repeated three times independently. Data are presented as mean ± SD. ∗*p* < 0.05, ∗∗*p* < 0.01, ∗∗∗*p* < 0.001, and ∗∗∗∗*p* < 0.0001.

Although recent studies reported that ferroptosis played an important role in intervertebral disc degeneration, the relationship of ferroptosis with CEP has yet to be explored. Nrf-2 is a key regulator of ferroptosis (Wu et al., 2022). We next investigated whether ferroptosis participates in CEP degeneration and the role of Nrf-2 mediated ferroptosis regulation in the protect effect of CEP degeneration. As shown in Figures 7A,B, D, G, Western blot and immunofluorescence analysis showed notably decreased protein expressions of GPX4, SLC7A11 after TNF-α and TBHP treatment. In addition, MDA levels were increased and SOD activity was decreased after TBHP treatment (Figure 7E), which indicated that IVDD pathological conditions including inflammation and oxidative stress could promote endplate chondrocytes ferroptosis. Our *in vivo* experiments also showed that mice in the IVDD group exhibited decreased GPX4 expression (Figure 7I). ICA treatment led to notably increased protein levels of GPX4, FTH1, SLC7A11, and SOD activity, decreased MDA level in endplate chondrocytes stimulated with TNF-α and TBHP, which indicated that ICA inhibited endplate chondrocyte ferroptosis (Figures 7A,B, D, G). Immunohistochemistry assay of GPX4 in the IVDD mice model also showed that ICA administration inhibited endplate chondrocytes ferroptosis (Figure 7G). Nrf-2 siRNA was then synthesized and transfected into cells to investigate whether ICA inhibited ferroptosis *via* activating Nrf-2. As shown in Figures 7C, H, knockdown of the Nrf2 expression blocked the ICA-mediated upregulation of GPX4, FTH1, and SLC7A11, indicating that ICA decreased chondrocytes ferroptosis *via* Nrf-2 activation. These results indicated that ferroptosis was involved in CEP degeneration and ICA suppressed the TNF-α and H_2_O_2_-induced chondrocyte ferroptosis by activating the Nrf2/HO-1 signaling pathway.

## Discussion

Cartilage endplate degeneration and calcification could significantly affect the biomechanics and nutrient supply status of intervertebral disc, which is widely recognized as an important contributor to the onset and development of IVDD (3). Although ICA has been proven beneficial effects in cartilage chondrocytes and NP cells under physiological conditions, its effect and mechanism on endplate chondrocytes are poorly understood. In the present study, our *in vivo* and *in vitro* experiments demonstrated that ICA protected against CEP degeneration and IVDD development. ICA could activate the endplate chondrocytes mitophagy process and inhibit ferroptosis, which subsequently promote cell viability and inhibit endplate ECM degradation enzyme genes expression *via* activating the Nrf-2/HO-1 signaling pathway under pathological conditions.

Several clinical evidence reported that patients with endplate osteochondritis easily suffer low back pain and lumbar disc herniation (Rade et al., 2018). Recent *in vivo* and *in vitro* studies have demonstrated the essential role of endplate degeneration in the initiation and progression of IVDD, inhibiting CEP degeneration, and calcification *via* melatonin or antioxidants could ameliorate the IVDD process (Ding et al., 2014; Zhong et al., 2016). In the present study, we first investigated the protective effect of ICA in CEP degeneration *in vivo*. The IVDD mice model used in this study was established by transection of bilateral facet joints, which could cause intervertebral disc instability and CEP degeneration (Ao et al., 2019). As expected, mice in the IVDD group exhibited severe CEP degeneration and calcification with obvious decreased cartilage matrix and increased bony tissues that contained bone marrow and mineralized bone in the deep zone of cartilage endplate, while ICA administration not only inhibited the bony tissue formation in the CEP but also increased the amounts of extracellular matrix both in NP and CEP. These results demonstrated that ICA could inhibit CEP degeneration and IVDD development, thus providing new insights into the treatment of IVDD.

The pathogenesis of IVDD is very complex, and chondrocyte is the only cell type in the endplate. Various risk factors such as mechanical overload, injury, instability, postural bipedality, chemical or genetic disorders, and smoking could lead to CEP inflammation and chondrocytes oxidative stress, thus decrease the cell viability and promote CEP degeneration (Vergroesen et al., 2015; Dowdell et al., 2017). Several *in vivo* and *in vitro* evidence have shown that inflammation and oxidative stress are widely present in degenerated intervertebral discs and both contribute to the apoptosis and calcification in cells, including EPCs (Kang et al., 2020; Wang et al., 2020). Therefore, we tested the mechanism of ICA on CEP degeneration and chondrocytes apoptosis using both inflammatory mediators TNF-α and oxidants TBHP to induce CEP degeneration. In the present study, we demonstrated that ICA co-treatment inhibited TNF-α and TBHP induced chondrocytes apoptosis and ECM degradation. Also our results showed that chondrocytes hypertrophic and osteogenic markers COL10 and RUNX2 protein levels were both decreased after ICA treatment, which suggested that ICA could attenuate endplate calcification. Together, our *in vitro* results demonstrated that ICA could inhibit apoptosis and calcification under both inflammation and oxidative stress conditions, thus inhibiting CEP degeneration.

Oxidative stress may induce mitochondrial dysfunction and morphology destruction, which could in turn further lead to ROS overproduction and activate the mitochondrial apoptotic pathway *via* decreasing anti-apoptosis-related protein, Bcl-2, and promoting the release of mitochondrial apoptosis-related protein, Bax (He et al., 2018). Recently, mitophagy has attracted growing interest owing to its roles in maintenance of mitochondrial homeostasis *via* clearing damaged mitochondria. Alterations in the expression of mitophagy marker, Parkin could affect the mitochondrial homeostasis, which has been demonstrated to be associated with the pathogenesis of several diseases, including IVDD (22); however, its effect on endplate chondrocytes was not uniform. Kang Liang et al. reported that under pathological conditions, Parkin is recruited to the outer membrane of damaged mitochondria by PINK1, and cellular mitophagy is activated (Kang et al., 2020). However, there are also studies indicated that the autophagy process was inhibited during CEP degeneration and decreased Parkin protein contributes to the IVDD development (Zhang et al., 2018). Therefore, the regulation of mitophagy in CEP degeneration and its underlying mechanisms still need further investigation. In the present study, we found that endplate chondrocytes exhibited decreased Parkin expression after TNF-α and TBHP treatment, along with ROS overproduction, mitochondrial dysfunction, and apoptosis, while ICA treatment promoted the expression and colocalization of Parkin, LC3 with mitochondria, indicating that mitophagy was activated.

The transcription factor Nrf-2 is a master regulator of cellular redox homeostasis and is widely involved in various cellular functions and contributes to cytoprotection against environmental stress and oxidative damage. In recent studies, reduced levels of Nrf-2 were found in degenerated intervertebral discs, and activating Nrf-2 has been demonstrated to be a promising treatment strategy for preventing IVDD development (Kang et al., 2020). Several recent studies suggested that Nrf2 was involved in the fine-tuning of the autophagic process in response to oxidative stress and could directly regulate the mitophagy process *via* regulating Parkin protein expression. In the present study, we demonstrated that Nrf-2/HO-1 played important roles in the protective effect of ICA in CEP degeneration; inhibition Nrf-2 not only partly abrogated the protective effect of ICA under TNF-α or TBHP stimulation but also inhibited Parkin protein expression and mitophagy process.

The endplate chondrocytes apoptosis and the subsequent reduction of extracellular matrix production is the direct cause of CEP degeneration. Ferroptosis is a newly identified type of programmed cell death (PCD), which is characterized by the production of reactive oxygen species (ROS) in the iron-mediated Fenton reaction. Ferroptosis is associated with many diseases, such as Parkinson’s syndrome, malignancies, and neurodegenerative diseases (Li et al., 2020). Recent studies indicated that various IVDD risk factors, such as oxidative stress, inflammation, abnormal lipid, and amino acid metabolism could promote chondrocytes ferroptosis and decrease the cell viability (Stockwell et al., 2017). However, the role of ferroptosis in CEP has rarely been reported. Moreover, inhibiting oxidative stress injury and cell apoptosis *via* maintaining mitochondrial homeostasis, the key iron storage proteins ferritin light and heavy chains (FTL/FTH1) and ferroptosis marker, GPX4 are regulated by Nrf2 (17). Therefore, we explored whether ferroptosis is activated during the IVDD development and whether ICA could inhibit ferroptosis *via* activating Nrf-2. Our results showed that both TNF-α and TBHP significantly promoted endplate chondrocytes ferroptosis. Similar results were obtained by our *in vivo* experiments that GPX4 protein expression significantly decreased in the IVDD group, indicating ferroptosis was involved in IVDD development under pathological conditions. ICA administration reversed TNF-α and TBHP induced GPX4, FTH1, and SLC7A11 proteins downregulation and MDA upregulation, which indicated that ICA could inhibit endplate chondrocytes ferroptosis under pathological conditions. Moreover, we found that knockdown of Nrf-2 partly abolished the protective effect of ICA in chondrocytes ferroptosis. These results suggest that ferroptosis is involved in the CEP degeneration, and ICA could inhibit ferroptosis *via* activating the Nrf-2/HO-1 pathway.

**FIGURE 8:**
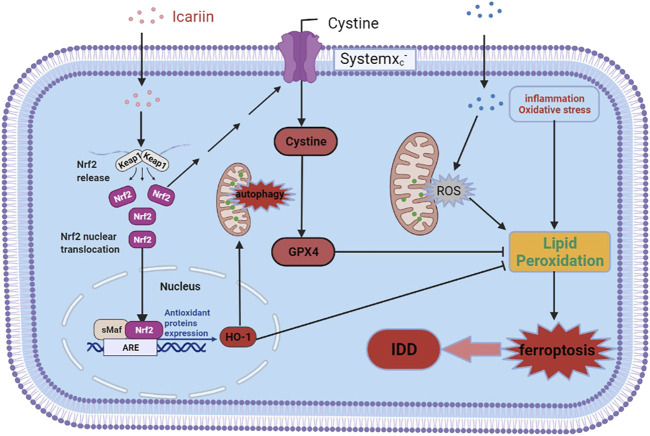
Schematic diagram of ICA-mediated protective effects on chondrocytes. ICA suppressed the TNF-α and H_2_O_2_-induced inflammation, oxidative stress, and ferroptosis by activating the Nrf2/HO-1 signaling pathway.

In conclusion, our study demonstrated that ICA could protect against cartilage endplate degeneration and calcification under IVDD pathological conditions, and the associated mechanism may be related to Nrf-2/HO-1 mediated mitophagy activation and ferroptosis inhibition, thus alleviated redox imbalance and mitochondrial dysfunction and eventually improved cell survival. The detail molecular mechanisms of ICA in CEP on IVDD are illustrated in Figure 8. Our results suggest that ICA may be a potentially effective medicine for IDD prevention and treatment.

## Data Availability

The original contributions presented in the study are included in the article/Supplementary Material; further inquiries can be directed to the corresponding authors.
