# Supplementary material for: Icariin protects vertebral endplate chondrocytes against apoptosis and degeneration via activating Nrf-2/HO-1 pathway
Source: Front Pharmacol. 2022 Sep 13;13:937502. doi: 10.3389/fphar.2022.937502 (PMC9513224; doi:10.3389/fphar.2022.937502)
Supplement: Supplementary file 1 [file DataSheet2.docx]

**Raw Data：**

https://www.jianguoyun.com/p/DVj8PwgQyYfLChjBz8AEIAA
