# Supplementary material for: Icariin protects vertebral endplate chondrocytes against apoptosis and degeneration via activating Nrf-2/HO-1 pathway
Source: Front Pharmacol. 2022 Sep 13;13:937502. doi: 10.3389/fphar.2022.937502 (PMC9513224; doi:10.3389/fphar.2022.937502)
Supplement: Supplementary file 2 [file DataSheet1.docx]

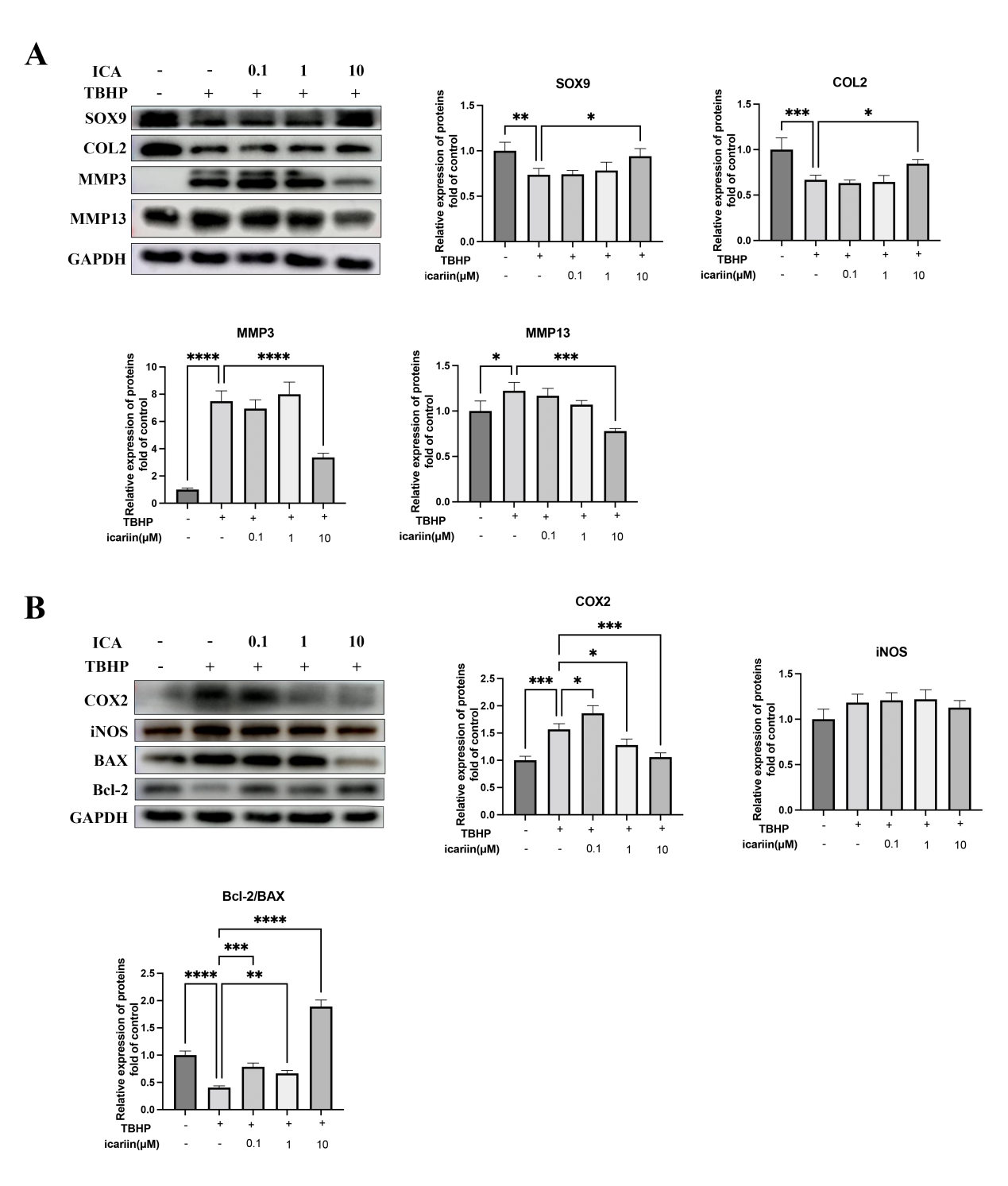


Supplementary Figure 1. The effect of ICA in TBHP induced nucleus pulposus cells degeneration and apoptosis. (A)Human NP cells were isolated and treated with TBHP(100μM)and ICA(0.1, 1, 10μM) for 24h and western blot was conducted to examine the protein levels of COL2, SOX9, MMP3and MMP13. The band density of COL2, SOX9, MMP3 and MMP13 was quantified and normalized to control. (B) **N**ucleus pulposus cells were treated with TBHP(100μM)and ICA(0.1, 1, 10μM) for 24h and western blot was conducted to examine the protein levels of COX2, iNOS, Bax and Bcl-2. The band density of COX2, iNOS, the ratio of Bcl-2/BAX was quantified and normalized to control.Data are presented as mean ± SD. ∗P < 0.05, ∗∗P < 0.01, ∗∗∗P < 0.001, and ∗∗∗∗P < 0.0001.


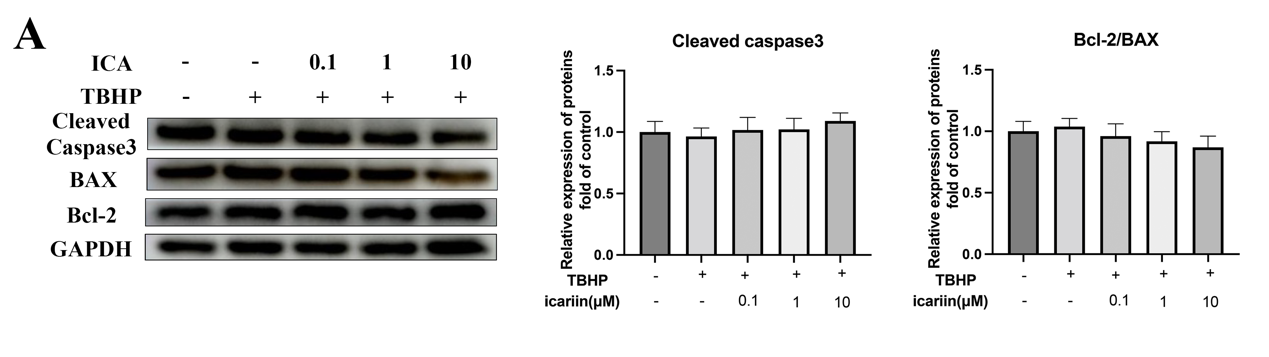


Supplementary Figure 2. The effect of ICA in TBHP induced annulus fibrosus cells apoptosis. Human annulus fibrosus cells were isolated and treated with TBHP(100μM)and ICA(0.1, 1, 10μM) for 24h and western blot was conducted to examine the protein levels of Cleaved caspase 3, BAX and Bcl-2. The band density of cleaved-caspase 3, the ratio of Bcl-2/BAX was quantified and normalized to control.Data are presented as mean ± SD.


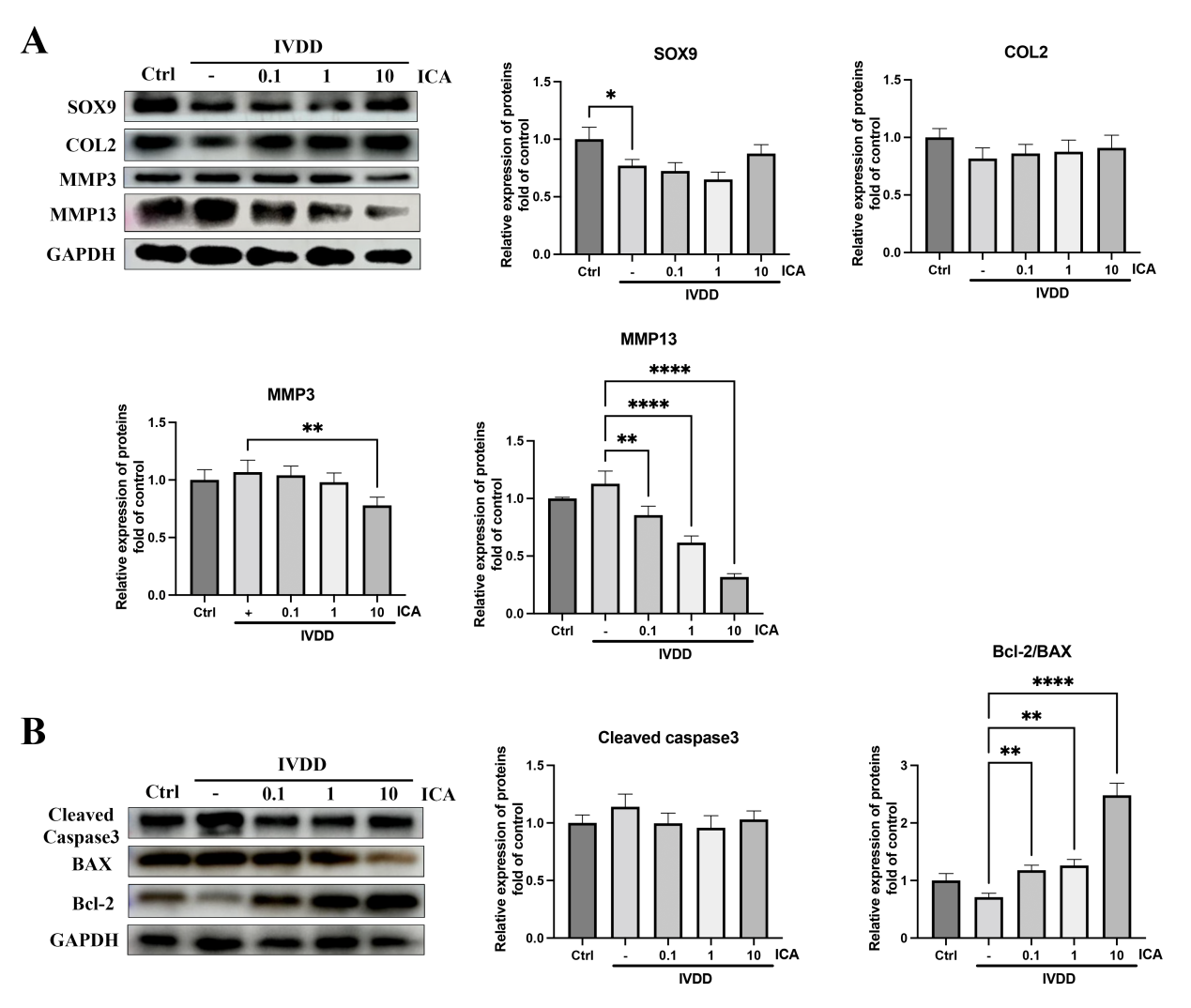


Supplementary Figure 3.CEP chondrocytes from IVDD model mice were isolated and treated with ICA(0.1, 1, 10μM), CEP chondrocytes from normal mice were isolated as control. Western blot was conducted to examine the protein levels of COL2, SOX9, MMP3and MMP13. The band density of SOX9, COL2, MMP3 and MMP13 was quantified and normalized to control. (B) Western blot was conducted to examine the protein levels of Cleaved-Caspase3, Bax and Bcl-2. The band density of Cleaved-Caspase3, the ratio of Bcl-2/BAX was quantified and normalized to control.Data are presented as mean ± SD. ∗P < 0.05, ∗∗P < 0.01, and ∗∗∗∗P < 0.0001.
